# Lingual Ischemia as a Rare Complication of Terlipressin Therapy: A Case Report

**DOI:** 10.7759/cureus.111185

**Published:** 2026-06-20

**Authors:** Joana Luís, Patrícia Varela Ramos, Ana M Oliveira, Ana Margarida Araújo, Rafael Curto

**Affiliations:** 1 Intensive Care Unit, Unidade Local de Saúde (ULS) Estuário do Tejo, Vila Franca de Xira, PRT

**Keywords:** hepatorenal syndrome, intensive care, ischemic complications, lingual ischemia, terlipressin

## Abstract

Terlipressin is a synthetic analogue of vasopressin commonly used in the management of hepatorenal syndrome due to its potent and selective vasoconstrictive effects on the splanchnic circulation. Although generally well tolerated, it is associated with rare but potentially severe ischemic complications. This report describes a 76-year-old woman who was admitted with asthenia and progressive abdominal distension. Her medical history included hypertension, hypercholesterolemia, obesity, chronic atrial fibrillation, chronic heart failure, chronic kidney disease, and metabolic dysfunction-associated steatotic liver disease with large-volume ascites. On admission, she was hypotensive and disoriented, with laboratory findings consistent with hepatic and renal dysfunction. She was diagnosed with spontaneous bacterial peritonitis and treated with ceftriaxone. Due to hepatorenal syndrome and grade 4 hepatic encephalopathy, she was transferred to the ICU and received human albumin (1 g/kg/day for two days) and terlipressin (6 mg/day). On the fourth day of terlipressin administration, the patient developed rapidly progressive tongue ischemia, prompting immediate discontinuation of the drug. Despite supportive measures, her condition deteriorated, leading to refractory shock and death on the sixth day of hospitalization. Lingual necrosis is a very rare ischemic complication associated with terlipressin use. Prompt recognition is essential for immediate drug withdrawal and potential prevention of fatal outcomes.

## Introduction

Terlipressin, a synthetic long-acting analogue of vasopressin, is widely used for the treatment of hepatorenal syndrome and variceal bleeding [1,2]. Its pharmacological activity primarily affects vasopressin type I (V1) receptors and is mediated by vasoconstriction of the splanchnic circulation, reducing portal venous pressure, increasing systemic blood circulation and effective mean arterial blood pressure, and improving renal perfusion [2]. It is generally considered a safe drug with mild adverse effects. However, despite its clinical benefits, terlipressin may cause ischemic complications in fewer than 5% of patients [2]. These events can affect multiple vascular territories, including the skin, gastrointestinal tract, extremities, and, very rarely, the tongue due to its rich vascular supply [1,3,4].

Lingual ischemia is an exceptionally uncommon adverse effect of vasopressors, especially in critically ill patients with end-organ hypoperfusion [4]. Given its potential severity and the challenges of early diagnosis, awareness of this complication is crucial for intensive care physicians. We present a case of lingual necrosis following terlipressin therapy in a critically ill patient with hepatorenal syndrome.

## Case presentation

A 76-year-old woman was admitted to the emergency department with a one-week history of worsening asthenia, abdominal distension, nausea, and vomiting. Her medical history included hypertension, hypercholesterolemia, obesity, chronic atrial fibrillation, chronic heart failure with preserved ejection fraction, chronic kidney disease (stage 3b), and metabolic dysfunction-associated steatotic liver disease with large-volume ascites.

On admission, the patient was conscious, alert, and oriented to person but disoriented to time and place. Her mucous membranes were pale and anicteric. She was tachypneic while breathing room air, with an oxygen saturation (SpO₂) of 98%. Blood pressure was 84/65 mmHg, and heart rate was 105 bpm. Cardiopulmonary auscultation was unremarkable. Abdominal examination revealed marked distension due to tense ascites, with tenderness on both superficial and deep palpation. There was bilateral lower-limb edema with grade 4+ pitting. Arterial blood gas analysis showed a normal pH (7.40), pCO₂ of 22.9 mmHg, pO₂ of 107 mmHg, HCO₃⁻ of 13.9 mmol/L, and lactate of 2.14 mmol/L. The laboratory findings are summarized in Table 1.

**Table 1:** Laboratory evaluation at admission

Blood analysis	Results (units)	Normal values
Hemoglobin	13.3 g/dL	12.0-15.0 g/dL
Platelets	331,000/µL	150,000-400,000/µL
International normalized ratio	1.83	0.8-1.2
Prothrombin time	21.4 seconds	11-13 seconds
Creatinine	2.98 mg/dL	0.55-1.02 mg/dL
Urea	139 mg/dL	19-49 mg/dL
Alanine aminotransferase	22 U/L	7-56 U/L
Gamma-glutamyl transferase	74 U/L	9-48 U/L
Alkaline phosphatase	133 U/L	44-147 U/L
Albumin	2.49 g/dL	3.5-5.0 g/dL
Total bilirubin	1.07 mg/dL	0.1-1.2 mg/dL
Ammonia	258 µmol/L	15-45 µmol/L
C-reactive protein	9.59 mg/dL	<0.34 mg/dL
Lactate	2.14 mmol/L	<2 mmol/L

Diagnostic and evacuatory paracentesis (4 L) revealed findings consistent with spontaneous bacterial peritonitis (ascitic fluid slightly turbid; leukocytes 1,060 cells/µL, including 391 polymorphonuclear cells; albumin 1.23 g/dL; total protein 3 g/dL; lactate dehydrogenase 88 U/L; amylase 32 U/L; pH 7.78). Blood cultures were obtained, and ceftriaxone was initiated.

Hemodynamic assessment revealed hypotension with suspected relative hypovolemia in the context of large-volume ascites and sepsis. Although invasive hemodynamic monitoring was not available, clinical findings suggested reduced effective arterial blood volume. Point-of-care ultrasound was used to monitor volume status, and no signs of volume overload (intravascular volume overload and/or pulmonary edema) were found that could potentially contraindicate albumin administration. Given severe hepatic encephalopathy, lactulose and rifaximin were also initiated.

The patient exhibited clinical deterioration, with worsening mental status (responsive only to painful stimuli, without spontaneous eye opening) and a generalized seizure episode. Arterial blood gas analysis revealed severe metabolic acidosis (pH 7.06, pCO₂ 30.7 mmHg, pO₂ 203 mmHg, HCO₃⁻ 9 mmol/L) and elevated lactate of 4 mmol/L. Blood pressure was 114/74 mmHg, and heart rate was 115 bpm. The patient was intubated and transferred to the ICU. A diagnosis of grade 4 hepatic encephalopathy and type 1 hepatorenal syndrome was established.

Severity scores were calculated to better characterize prognosis. The patient had an estimated Model for End-Stage Liver Disease (MELD) score of 23 points (19.6% estimated 3-month mortality) and Child-Pugh class C, consistent with advanced liver disease [5]. On ICU admission, the Sequential Organ Failure Assessment (SOFA) score was 5 (33.3% mortality), reflecting multiorgan dysfunction.

She received human albumin (1 g/kg/day for two days) and terlipressin at 6 mg/day (continuous infusion), as supported by the guidelines [6,7]. The patient had no known allergies to drugs or foods. No additional vasopressors (including norepinephrine, vasopressin, or dopamine) were administered during the ICU stay, and terlipressin was the sole vasoactive agent used.

**Figure 1:**
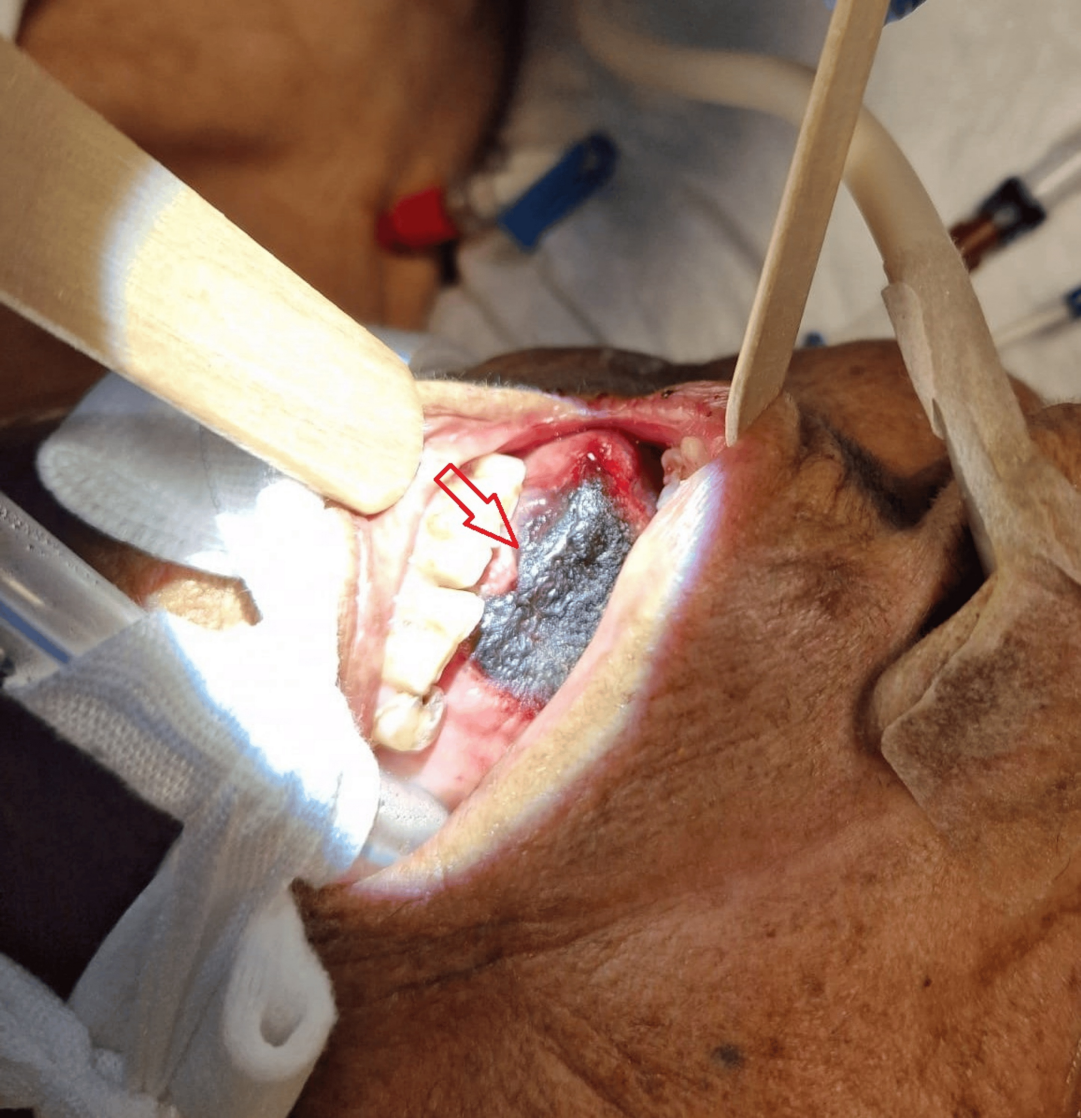
Lingual ischemia characterized by bilateral tongue necrosis with rapid progression from the distal to the proximal segment

**Figure 2:**
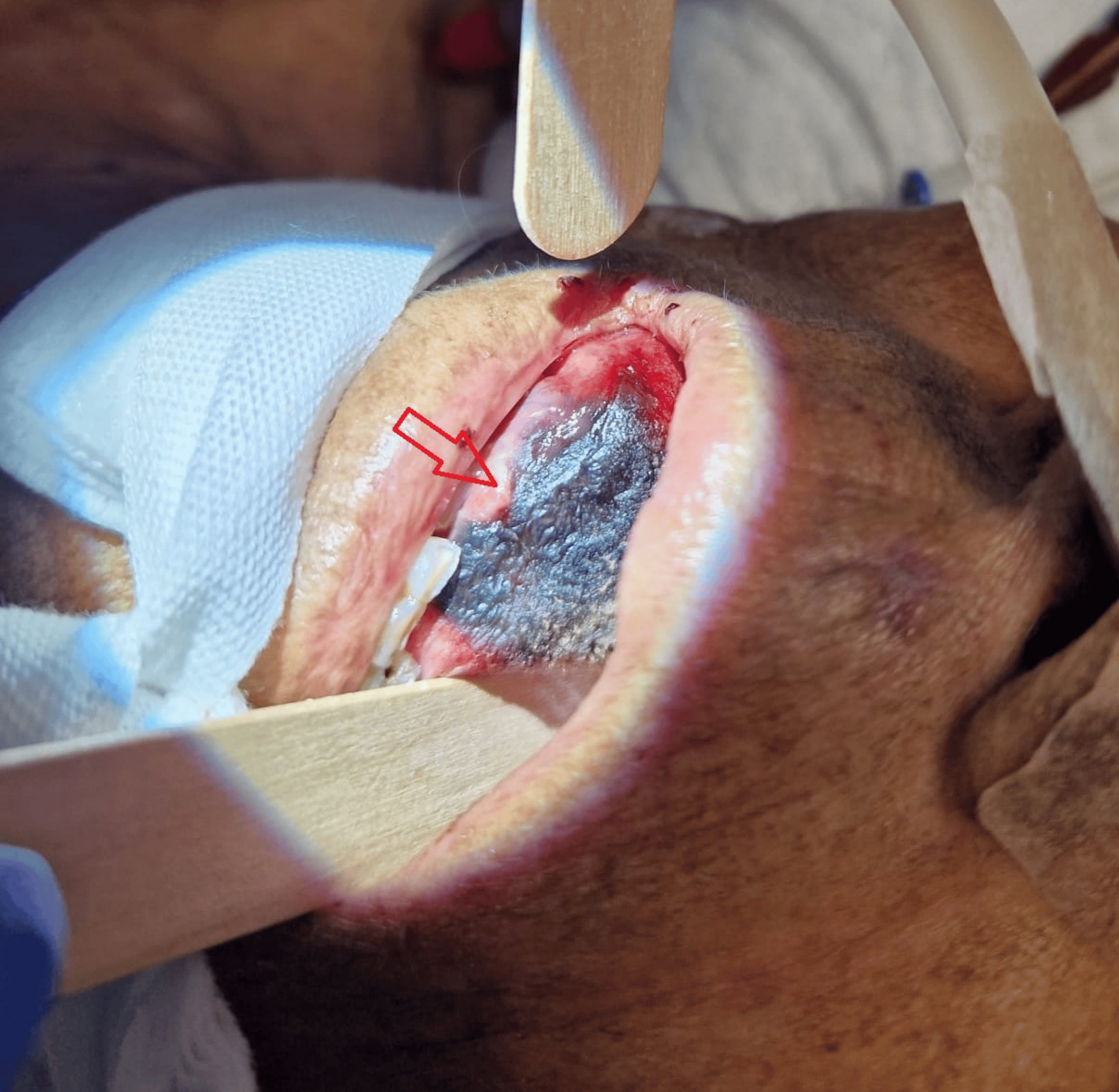
Lingual necrosis

On the fourth day of terlipressin therapy, she developed rapidly progressive lingual ischemia, characterized by dark discoloration and necrotic changes, documented by photographic evidence (Figures 1, 2). Informed consent was obtained from the patient’s legal representative. There was no opportunity to perform a skin biopsy. Terlipressin was immediately discontinued.

Despite instituted therapy, including intravenous albumin for volume expansion, broad-spectrum antibiotic therapy (ceftriaxone) for infection control, and lactulose and rifaximin for hepatic encephalopathy, the patient showed progressive clinical deterioration. There was worsening renal function and ascites consistent with refractory hepatorenal syndrome, as well as persistent severe encephalopathy (Glasgow Coma Scale score of 3) with seizures. Metabolic acidosis and elevated inflammatory markers persisted despite treatment. The patient developed multiorgan dysfunction with signs of poor peripheral perfusion, with a peak lactate of 10 mmol/L. The clinical course culminated in death on the sixth day of hospitalization due to multiorgan failure secondary to hepatic decompensation, as shown in Table 2.

**Table 2:** Clinical timeline

Day	Events	Treatment	Key findings
0	Admission	Ceftriaxone + albumin bolus	Hypotension, renal dysfunction, spontaneous bacterial peritonitis
1	ICU admission	Albumin + terlipressin started	Glasgow Coma Scale 7, metabolic acidosis, orotracheal intubation
2	Clinical deterioration	Continued therapy	Persistent renal dysfunction and encephalopathy
4	Lingual ischemia onset	Terlipressin stopped	Tongue necrosis
5	Refractory shock	Supportive care	Rising lactate
6	Death	Comfort measures	Multiorgan failure

## Discussion

Terlipressin is a synthetic analogue of vasopressin commonly used in the management of hepatorenal syndrome due to its potent and selective vasoconstrictive effects on the splanchnic circulation [8,9]. It is enzymatically converted in the circulation into lysine vasopressin following endothelial peptidase cleavage of the N-triglycyl residue, which accounts for its slow release of vasopressin, prolonged activity, fewer side effects, and a more favorable safety profile [1,2,8,9]. This mechanism prolongs its half-life (estimated plasma half-life of approximately six hours, in contrast to six minutes for vasopressin), enabling intermittent bolus administration without the need for continuous infusion, unlike vasopressin in clinical practice [2,8,9]. Thirty minutes after terlipressin administration, a significant increase in mean arterial pressure and systemic vascular resistance is observed, along with a reduction in heart rate, cardiac output, hepatic venous portal pressure gradient, and portal venous blood flow [2]. There is evidence that this drug is superior to placebo in reducing renal vasoconstriction and consequently improving renal function in patients with hepatorenal syndrome [2].

Terlipressin demonstrates strong affinity for V1 receptors located in the smooth muscle of blood vessels, particularly within the splenic, renal, myometrial, bladder, adipose, and cutaneous vascular beds. This vasoconstrictive effect has been associated with impaired tissue oxygenation secondary to microcirculatory failure, ultimately resulting in ischemia and necrosis. In addition, activation of endothelial V2 receptors promotes the release of thrombogenic mediators that enhance platelet aggregation, contributing to distal microcirculatory obstruction through thrombus formation [8]. Evidence suggests that obesity (present in our patient) increases vulnerability to terlipressin-induced skin necrosis, likely due to mechanical stretching of the abdominal and lower-limb skin, which increases the microvascular perfusion area and predisposes to ischemia [10]. Additional contributing factors include hypovolemia, concurrent use of other vasopressors, ischemic disease, ascites, continuous terlipressin infusion, obesity, and spontaneous bacterial peritonitis [2,4,11], the latter of which were present in our case.

Although terlipressin has a preferential effect on the splanchnic circulation, systemic vasoconstrictive effects can occur, particularly in predisposed patients with advanced liver disease, renal impairment, or hemodynamic instability, as in our case. The most frequently reported adverse reactions include pallor, headache, abdominal pain, bradycardia, and hypertension [8,9,11]. More severe but uncommon complications, such as myocardial infarction, ischemic colitis, and skin necrosis, have also been documented [8,9]. Published case reports usually describe ischemic involvement of the abdominal wall, thighs, legs, toes, scalp, scrotum, or esophagus. Less frequently, ischemic lesions have been observed in the feet, breasts, back, forearms, and tongue [2,9]. Further analysis indicates that skin necrosis typically develops within two to five days after initiation of terlipressin, most frequently in patients receiving bolus dosing (85.7%) [11]. Our case followed a similar timeline, with tongue necrosis emerging on the fourth day of continuous infusion. Reported cases suggest no clear correlation between the dose or duration of terlipressin administration and the severity of ischemia, emphasizing individual susceptibility [8,11]. Some authors have suggested that severe adverse effects are independent of both administration method (infusion or bolus) and daily or cumulative dose [11]; however, others have proposed that continuous infusion may reduce adverse effects by allowing lower total drug exposure [2,8,9]. According to Zhou et al., vasopressin-related serious adverse events in patients with septic shock were independent of vasopressin dose [8].

Pharmacogenomics may also play a role in predicting and preventing terlipressin-induced serious adverse events [12], although further research is needed. The differential diagnosis of lingual necrosis in this patient includes severe systemic hypoperfusion (shock), vasopressor-induced vasoconstriction, thromboembolic events, and local mechanical factors such as endotracheal tube compression. Severe shock was considered; however, the onset of lingual ischemia preceded refractory shock, occurred within the timeframe described in the literature, and developed in the absence of other vasopressors [8]. Thromboembolic causes could not be definitively excluded, although there was no clinical evidence of embolic phenomena in other vascular territories. Mechanical compression was considered less likely given the bilateral and progressive pattern of necrosis, which is more suggestive of a systemic vascular process. Therefore, terlipressin-induced vasoconstriction remains the most plausible contributing factor.

Lingual ischemia is a particularly rare manifestation, with only isolated cases described in the literature [2,4,8]. The tongue has a rich vascular supply from the bilateral lingual arteries and branches of the facial and pharyngeal arteries, making ischemia uncommon; however, systemic vasoconstriction and microvascular compromise can overcome these protective mechanisms [4,8], particularly in critically ill patients. In addition, mechanical pressure from the endotracheal tube may contribute to tongue ischemia by promoting edema and further reducing lingual perfusion [4].

In our case, the patient presented with bilateral tongue necrosis with rapid progression from the distal to the proximal segment (Figures 1, 2), which was less consistent with local pressure from an endotracheal tube and more suggestive of a systemic process. The history of hypertension and hypercholesterolemia may indicate underlying atherosclerosis, which could have contributed to ischemia [3].

The presence of ischemic necrosis of the tongue may be associated with a poor prognosis [4,10]. Early recognition of ischemic signs such as discoloration, pain, or ulceration should prompt immediate discontinuation of terlipressin [9,11]. Supportive care and close monitoring are essential, as progression to necrosis can be rapid and irreversible. Management involves discontinuation of terlipressin and supportive care to promote restoration of blood flow and tissue healing [8,10,11].

Despite extensive investigation into the mechanisms of terlipressin-associated ischemic injury, the precise pathophysiology remains incompletely understood [10]. Tongue necrosis is a rare condition with a broad differential diagnosis, including systemic vasculitis, disseminated intravascular coagulation, thromboembolic events, and profound shock-related hypoperfusion. In this case, based on the temporal relationship between terlipressin administration and onset of tongue necrosis, together with the absence of a more likely alternative explanation, the adverse reaction was classified as “probable/likely” [13].

This case highlights the importance of clinician awareness regarding rare ischemic complications of terlipressin, especially in critically ill patients. Nevertheless, most fatalities are attributable not to necrosis itself but to complications of advanced liver disease, which is commonly observed in patients receiving terlipressin [9], as in our case.

## Conclusions

This case highlights the complexity of advanced liver disease complicated by hepatorenal syndrome, severe hepatic encephalopathy, and multiorgan dysfunction. Despite timely implementation of recommended therapies, including albumin and terlipressin, the clinical course was rapidly progressive and fatal, reflecting the high mortality in critically ill patients with cirrhosis. Although terlipressin remains a cornerstone in the management of hepatorenal syndrome, it may be associated with rare but severe ischemic complications, such as lingual necrosis, particularly in patients with hemodynamic instability, infection, and impaired microcirculation. However, the multifactorial nature of ischemic events precludes definitive attribution to terlipressin alone.

This report emphasizes the importance of severity assessment, hemodynamic monitoring, and early evaluation of treatment response in advanced cirrhosis. Continuous reassessment is essential to balance the benefits of vasoconstrictor therapy against potential adverse effects and to facilitate prompt recognition of uncommon complications. Ultimately, outcomes in hepatorenal syndrome are driven by the severity of underlying liver disease and multiorgan failure. Further research is needed to identify risk factors for terlipressin-associated ischemic complications and to optimize management strategies in this high-risk population.
